# “Mirror” Method to Estimate Mutagenic Activity of DNA Lesions

**DOI:** 10.3390/mps1030032

**Published:** 2018-08-27

**Authors:** Leonid V. Gening, Oleg V. Shevchenko, Konstantin Y. Kazachenko, Vyacheslav Z. Tarantul

**Affiliations:** Institute of Molecular Genetics, Kurchatov Sq. 2, Moscow 123182, Russia; olegshevtschenko@yandex.ru (O.V.S.); konstantinkazach@yandex.ru (K.Y.K.); tarantul@img.ras.ru (V.Z.T.)

**Keywords:** DNA lesions, 7,8-dihydro-8-oxoguanine, 8-oxoG, mutagenic activity, method of detection

## Abstract

We propose an improved method for detecting mutations that arise in DNA due to misincorporations of deoxyadenosine-5′-monophosphate (dAMP) opposite 7,8-dihydro-8-oxoguanine (8-oxoG). The method is based on the synthesis of complementary chains (“mirror” products) using a template containing 8-oxoG. The misincorporation of dAMP in the “mirror” product forms *EcoRI* sites. The restriction analysis of double-stranded DNAs obtained by PCR of “mirror” product allows quantification of the mutagenesis frequency. In addition, single-strand conformational polymorphism (SSCP) analysis of the single-stranded “mirror” products shows that different DNA polymerases only incorporate dA or dC opposite 8-oxoG. The proposed approach used in developing this technique can be applied in the study of other lesions as well, both single and clustered.

## 1. Introduction

It is known that tens of thousands of lesions occur daily in each human cell [1]. Obviously, the development of such lesions into fully realized mutations is one of the key moments in the pathogenesis of numerous diseases [2,3]. The ability to study the influence of various factors on such lesion development will allow the design of a new approach in the prevention and treatment of various diseases.

Currently, several methods are used to assess the mutagenic potential of a specific DNA lesion, with the most concerning lesions produced by oxidative stress. One such lesion, 7,8-dihydro-8-oxoguanine (8-oxoG), is particularly important because it is widely present in DNA [4,5]. To study the mutagenic effect of 8-oxoG, Shibutani et al. [6] performed a chain extension reaction in 1991, using an 8-oxoG-containing template and various DNA polymerases. Having electrophoretically separated the reaction products, they determined the potentially mutagenic incorporation of deoxyadenosine-5′-monophosphate (dAMP) as the sole possible misincorporation opposite 8-oxoG. Today, the primer extension assay, with four parallel DNA-dependent polymerase reactions carried out simultaneously (each of them with only one of the four types of nucleoside triphosphate (dNTP)), is usually performed in order to determine which nucleotide is incorporated opposite a certain lesion [7,8,9]. However, in all of those experiments, no separation of the primer extension reaction products was performed, and therefore no conclusion can be drawn as to whether those lesions cause the misincorporation of only one type of a nucleotide and do not generate other mutations as well.

Thus, the approach used by Shibutani et al. [6] seems more preferable since it not only shows the incorporation of an incorrect nucleotide opposite the introduced DNA lesion, but also detects mutations within the complementary chain and allows separation of the mutant and non-mutant molecules. Unfortunately, separating short single-strand DNA oligonucleotides having the same length in a denaturing polyacrylamide gel is a rather complicated task. This might explain why the approach is not employed by most researchers.

In this work, we propose a different approach for estimating the mutagenic potential of DNA lesions (using 7,8-dihydro-8-oxoguanine (8-oxoG) as an example). To do so, we synthesized DNA strands complementary to the lesion-containing template via primer extension reaction. The newly synthesized strands were denoted as “mirror” products, and further investigation of those products allowed the detection of mutations caused by 8-oxoG. The advantage provided by this approach is that the “mirror” products (DNA strands complementary to the starting template) may contain not only a substitution mutation opposite the lesion, but other sorts of mutations as well, either opposite the lesion or somewhere else in the strand. Each of those mutations will produce different nucleotide sequences, allowing their separation and subsequent identification, making for more informative results.

## 2. Materials and Methods

***DNA polymerases***. We used the catalytic core of the human polymerase ι (Pol ι), 420 amino acids long, fused with glutathione-*S*-transferase (GST) at the N end. The enzyme was produced in yeast using the expression yeast vector obtained and purified as described earlier [10]. The purified human polymerase κ (Pol κ) preparation used in this work was produced and isolated from *Escherichia coli* [11]. Klenow fragment (KF) was purchased from MBI Fermentas (Waltham, MA, USA) and contained 5 U/mL. Polymerase λ (Pol λ) was provided by O.I. Lavrik, Institute of Chemical Biology and Fundamental Medicine, Siberian Branch of the Russian Academy of Science. All enzymes were >95% pure.

***Templates for “mirror” product synthesis****.* The 8-oxoG (X) containing template (***AXT***) had the following nucleotide sequence:

5′-GGGATCCTGCTGCCATAGGA***AXT***CTTGATTGGAAAGTCGACCTGC-3′.

Two control templates were used. One (***ATT***) contained T in place where AXT had 8-oxoG:

5′-GGGATCCTGCTGCCATAGGA***ATT***CTTGATTGGAAAGTCGACCTGC-3′.

The other (***AGT***) contained G in place where AXT had 8-oxoG:

5′-GGGATCCTGCTGCCATAGGA***AGT***CTTGATTGGAAAGTCGACCTGC-3′.

***Primers****.* Two following oligodeoxyribonucleoties were used as reverse (RP) and direct (DP) primers:

5′-GCAGGTCGACTTTCCAATCAA-3′ (RP),

5′-CY3-GGGATCCTGCTGCCATAG-3′ (DP).

***Synthesis of the “mirror” products****.* The reaction mixture (overall volume of 20 μL) contained 5 nM the AXT (or either control ATT or AGT) template, a 10-fold excess of DNA polymerase to assure full-length primer extension (0.5 nM KF, 18 nM Pol ι, 10 nM Pol κ, or 15 nM Pol λ), 10 nM RP, 0.05 M tris-HCl pH 8.3, 1.0 mM MgCl_2_, and the mixture of 100 μM of each of the four dNTP. The reaction was carried out at 37 °C for 20 min. Afterward, the reaction mixture was incubated at 95 °C for 5 min to inactivate the DNA polymerase of interest.

***Asymmetric PCR*** was carried out in the mixture with the overall volume of 50 μL, and it contained 5 μL of the “mirror” product, 500 nM DP, 5 nM RP, and 25 μL of 2X DreamTaqMasterMix (Thermo Scientific, Waltham, MA, USA). PCR was carried under the following conditions: 95 °C for 20 s; 25 cycles of 95° for 20 s, then 60 °C for 20 s, then 72 °C for 20 s; 72 °C for 3 min.

***Second strand synthesis***. The reaction mixture (overall volume: 50 μL) contained 20 μL of the asymmetric PCR product, 25 μL of 2X DreamTaqMasterMix (Thermo Scientific), 500 nM RP. Second strand synthesis was carried out under the following conditions: 95 °C for 20 s; 2 cycles of 95° for 20 s, then 60 °C for 20 s, then 72 °C for 20 s; 72 °C for 3 min.

***EcoRI digestion*** was carried out in a 20 μL mixture containing 10 μL of the second strand synthesis product, 2 μL of 10X *EcoRI* buffer (Thermo Scientific), 5 U of *EcoRI* (Thermo Scientific). Digestion was carried out at 37 °C for 1 h.

***Electrophoresis of EcoRI digestion products*** was carried out in the dark at 10 °C in 20% polyacrylamide gel with Tris-borate buffer pH 7.5, using 25-centimeter-long glass, 18 mA current. Electrophoresis was performed for 4 h. The gel was scanned with Typhoon FLA 9500, the data were processed with ImageQuant™ v5.2 software (GE Healthcare Life Sciences, Little Chalfont, UK).

***Single-strand conformational polymorphism (SSCP) analysis***. Electrophoresis of the single-stranded products of asymmetric PCR was carried out in the dark at 10 °C in 20% polyacrylamide gel with Tris-borate buffer pH 7.5, using 25-centimeter-long glass, 18 mA current. Electrophoresis was performed for 20 h. The gel was scanned with Typhoon FLA 9500 (GE Healthcare Life Sciences), and the data were processed with ImageQuant™ v5.2 software (GE Healthcare Life Sciences).

## 3. Results and Discussion

**Figure 1:**
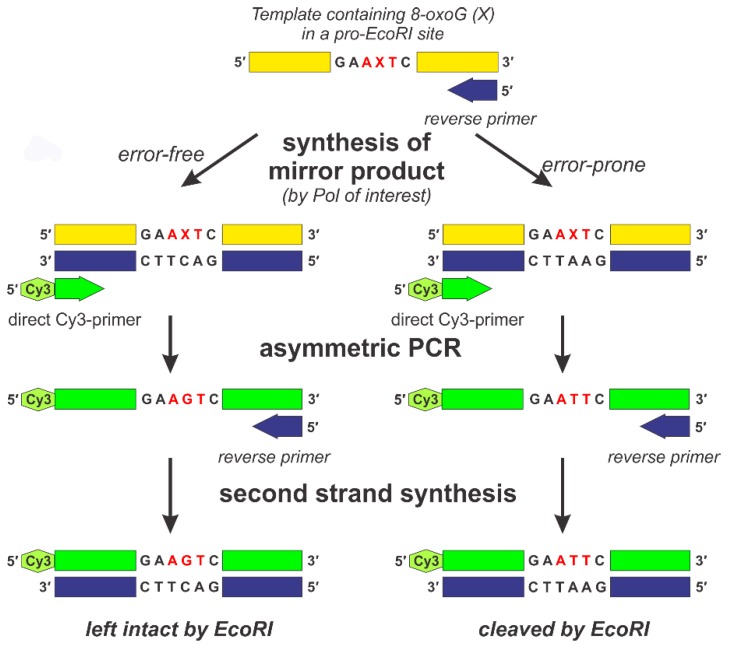
Schematic representation of the “mirror” method. A single-stranded DNA molecule containing 7,8-dihydro-8-oxoguanine (8-oxoG; X) is used as a template for synthesis of “mirror” products that exist as double-stranded hybrids with their original template. Depending on whether a particular “mirror” molecule was synthesized in an error-free or error-prone manner, it will contain either C or A opposite 8-oxoG, respectively. To eliminate any further influence of the 8-oxoG-containing template, asymmetric PCR is performed, yielding single-stranded DNA molecules containing either G or T in place of the original 8-oxoG. The former, when treated with *EcoR*I after conversion to double strand form, is left intact, whereas the latter is cleaved by *EcoR*I in its double strand form. Pol: DNA polymerase.

The principle of the approach in question is described as follows (see Figure 1). First, DNA “mirror” products are synthesized by different DNA polymerases (KF, Pol ι, Pol κ, and Pol λ) from an 8-oxoG (X)-containing DNA template using the RP complementary to the 3′-terminus of the template.

This template was designed to produce an *EcoRI* site when the misincorporation of dAMP opposite 8-oxoG occurs. After primer extension, DNA polymerases were inactivated, and the “mirror” products obtained in this step were used for further investigation.

The “mirror” products of the two control templates (ATT and AGT) were obtained by treating them with KF.

The obtained “mirror” products existed as hybrids with their templates. To avoid any further effect of the initial templates, we selectively amplified the “mirror” strand of the hybrids so that the amount of strands complementary to the “mirror” products was several orders of magnitude higher than that of any other DNA species present in the mixture. To do so, asymmetric PCR was performed using a DP containing a Cy3 marker. RPs were also added in this procedure. Since the amount of DP was 100 times that of RP, the “mirror” strand was the most kinetically favored as the template for asymmetric PCR in the early cycles. The minuscule amount of RP provided the opportunity for the single-strand DNA molecules obtained in the process to be amplified in the later cycles of PCR.

Then, single-strand DNA molecules were converted to double-strand products using RP and Taq-polymerase. The resulting mixtures were treated with *EcoRI*. 

**Figure 2:**
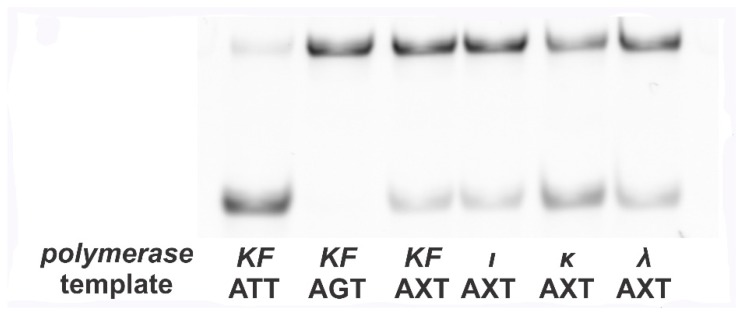
Electrophoretogram of the samples digested by *EcoRI*. Two controls demonstrate the fates of templates that contain G in place of 8-oxoG (no mutation, denoted as ***AGT***) and those that contain T in place of 8-oxoG (indicating mutation, denoted as ***ATT***). ***ATT*** is cleaved by *EcoRI*. Its only band is more mobile than that of ***AGT***, which in turn is left intact after treatment with *EcoRI*. The AXT template exhibits both bands for each polymerase used in the “mirror” product synthesis. The ratio between the bands’ intensities indicates mutagenic frequency in each case.

The products obtained from the control ATT template were completely cleaved by *EcoRI*, whereas those of the control AGT template did not undergo digestion (Figure 2). The products obtained from the AXT template by DNA polymerases of interest were cleaved only partially. Obviously, the ratio of cleaved to intact products depends on the misincorporation frequency of dAMP.

Quantitative analysis reveals that the deoxycytidine monophosphate (dCMP) to dAMP incorporation ratio is 0.089 ± 0.001 for KF, 0.088 ± 0.001 for Pol ι, 0.45 ± 0.02 for Pol κ, and 0.17 ± 0.01 for Pol λ. These numbers are almost equivalent to the results obtained by other researchers [6,7].

**Figure 3:**
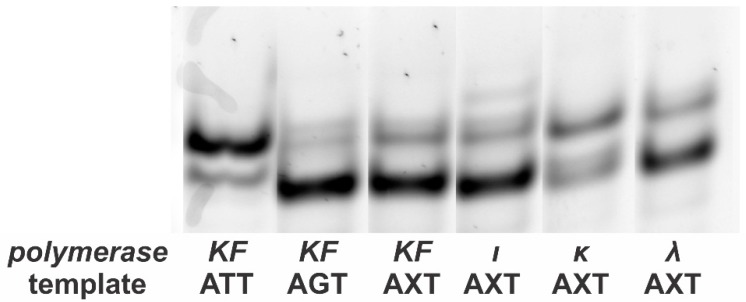
Single-strand conformational polymorphism (SSCP) analysis of the single-strand “mirror” products obtained as shown in Figure 1. Using ATT and AGT products obtained in asymmetric PCR as controls, one can differentiate between mutant molecules (containing T in place of 8-oxoG) and non-mutant molecules (containing G in place of 8-oxoG). It is evident from the electrophoretogram that only G→T transversion may occur when there is 8-oxoG present. The exception is Pol ι, which is well-known for its low fidelity. KF: Klenow fragment.

Our experiments included the consecutive incorporation of a much larger number of nucleotides than in other works [6,7]. Considering this, we decided to check our “mirror” products for any unspecified mutations. For that purpose, the single-stranded products of asymmetric PCR (Figure 3) were separated by electrophoresis in the same way as in the method that makes use of SSCP [12]. As shown in Figure 3, patterns formed by products of the two differing control templates were easily distinguished (the major product from the ATT template migrated more slowly). As for the lesion-containing AXT template, its patterns only exhibited bands characteristic of the two control templates, and the intensity ratio depended on the DNA polymerase used in the synthesis of the “mirror” products. These experiments proved that all the DNA polymerases used in the experiment strictly incorporated dCMP or dAMP. The exception is Pol ι, which exhibited an additional minor band. Its presence can be explained by the well-known low fidelity of this enzyme [13,14].

Thus, we propose a significant improvement to the primer extension method for the quantitative detection of mutations caused by 8-oxoG. In contrast to previous works by other authors, the approach we used allows not only the study of nucleotide incorporation opposite certain DNA lesions, but also the detection of mutations in the entire obtained strand. Observing both non-mutant and mutant DNA molecules and determining their ratio yields useful quantitative data. To do so, the two kinds can be distinguished by both restriction enzymes and electrophoretic mobility.

We believe that this approach may elucidate features of other DNA lesions as well, through devising templates containing such lesions. To find a suitable restriction enzyme for the analysis of a particular DNA lesion, it is a prerequisite to determine which mutations that lesion causes. Detecting and identifying its mutations can be achieved by the SSCP method [15]. It is based on the fact that different single-strand DNA molecules, depending on their nucleotide sequence, differ in their electrophoretic mobility through the non-denaturing polyacrylamide gel. It allows the separation of single-stranded DNA molecules up to several hundred nucleotides long differing by a single nucleotide and, as a result, the detection of point mutations in the genomes of various organisms. However, the SSCP method has not been applied to the study of the mutagenic potential of DNA lesions by the primer extension assay. In the past, we successfully employed it in the identification and separation of single-strand DNA aptamers of equal length, differing in their nucleotide sequence. Even scarcely present nucleotide sequences can be isolated for further amplification and sequencing [15]. The same procedure can be performed with mutant DNA molecules obtained after the DNA synthesis past various damaged sites, making the study of lesions other than 8-oxoG possible. Then, judging from the results, it should be possible to find a restriction enzyme suitable for further analysis.

The method proposed for the study of mutations produced during DNA synthesis past a certain lesion is rather promising as an in vitro method making use of relatively long DNA strands. Its closest analog is the Shibutani method based on the separation of short (18 nucleotides) DNA molecules of different nucleotide sequences. In this method, this can be achieved by using a restrictase or an SSCP method. In theory, this permits working with DNA molecules up to several hundred nucleotides long.

Currently, the mutagenic effects of various DNA lesions are also studied by in vivo methods. They involve the introduction of a lesion-containing DNA vector into living cells. Isolation of the said DNA after several cycles of in vivo replication and its further sequencing allows the mutagenicity of a lesion to be studied. Those methods are sometimes applied not only in the study of single lesions, but clustered lesions as well [16]. However, judging from the results of such research, the majority of the lesions are successfully repaired within a cell. When the investigation is performed in vitro using isolated DNA polymerases, the mutation frequency is significantly higher due to the lack of repair processes. This discrepancy highlights the existence of cellular systems preventing the mutagenicity of many oxidative DNA lesions. Research in the field of DNA repair will obviously benefit from the development of new and more advanced in vitro methods rendering the identification of DNA repair factors possible. The method we propose may serve as a prototype in the development of such methods.

## Abbreviations and Notations

8-oxoG	7,8-dihidro-8-oxoguanine
AXT	single-stranded DNA template containing 8-oxoG
ATT	single-stranded DNA template containing T in place of 8-oxoG
AGT	single-stranded DNA template containing G in place of 8-oxoG
DP	direct primer
GST	glutathione-*S*-transferase
KF	Klenow Fragment, *exo-*
Pol ι	DNA polymerase ι
Pol κ	DNA polymerase κ
Pol λ	DNA polymerase λ
RP	reverse primer
SSCP	single-strand conformational polymorphism
